# A zygote-based assay to evaluate intranuclear shuttling in *S. cerevisiae*

**DOI:** 10.1016/j.xpro.2021.100736

**Published:** 2021-08-12

**Authors:** Alan Michael Tartakoff

**Affiliations:** 1 Pathology Department and Cell Biology Program, Case Western Reserve University, Cleveland, OH 44106, USA

**Keywords:** Cell Biology, Genetics, Microscopy, Model Organisms

## Abstract

It is often necessary to learn whether macromolecules occupy a fixed place in cells. This protocol makes it possible to learn whether individual nucleolar proteins in *S. cerevisiae* remain in place or depart from and return to the nucleolus. The protocol uses early zygotes in which parental nucleoli are separate for at least one hour. The protocol demonstrates that the localization of many nucleolar proteins is in fact highly dynamic. Photobleaching is not required.

For complete details on the use and execution of this protocol, please refer to Tartakoff et al. (2021).

## Highlights

• The mobility of proteins within the yeast nucleus can be judged by constructing zygotes
• The assay requires minimal equipment other than a sensitive fluorescent microscope
• The protocol can be completed within one day once appropriate cells are available

## Graphical abstract

**Figure:**
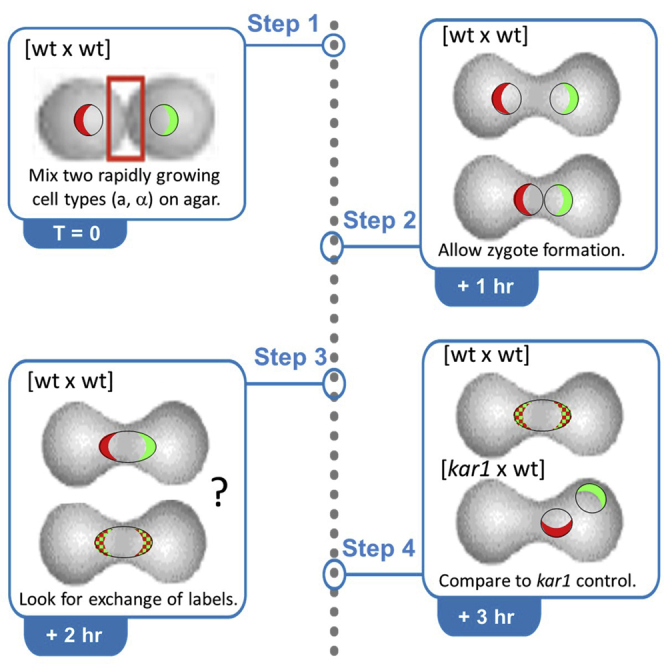

## Before you begin

Many intracellular structures that are spatially separate are highly dynamic. Individual components may move within the structure or repeatedly depart and return. Moreover, if multiple copies of "the same" structure are present, components may exchange between them. Ongoing departure and return of components provides a means by which their composition can be adjusted.

The procedure described here is based on yeast zygote formation. Similar procedures would be applicable in other situations in which heterotypic cell-cell fusion (and nuclear fusion) can be achieved. The procedure requires cells that express fluorescent proteins of interest but does not rely on photobleaching. Related procedures have been used to learn whether proteins shuttle in and out of the nucleus (Liu et al., 1996, Flach et al., 1994, Elion, 2002). We explain how this approach can be used to evaluate the dynamics of nucleolar proteins when - after nuclear fusion (karyogamy) - parental nucleoli remain separate for more than one hour (Tartakoff and Jaiswal, 2009). If a protein exhibits internucleolar shuttling during this time interval, we conclude that the protein normally departs from and returns to the nucleolus. In corresponding control crosses we learn whether any apparent shuttling occurs in the absence of karyogamy. It is striking that such impermanence is characteristic of several protein/RNA assemblages in the nucleus (Phair and Misteli, 2000).

### Recurrent procedures

1. Conduct all steps at either room temperature or at 30°C.
2. Use synthetic media for all experiments, as described in the Cold Spring Harbor Manual (Amberg et al., 2005).
3. Culture cells in 24-well plasticware dishes that are shaken.
4. Sediment cell cultures for 5 s at 5,000 **×** g in a table-top microfuge after transfer to Eppendorf tubes.
5. Use 0.5 mL volumes for all cell incubations and washes.

### Strain construction

**Timing: 2–10 days.**

To our knowledge, strains of distinct backgrounds can be used. We have used haploid strains derived from W303 or S288C. Strains of higher ploidy can presumably also be used if their mating types are appropriate.

6. Tag the protein(s) to be followed (e.g., the snoRNP protein,Sik1/Nop56, or the large ribosomal subunit assembly factor, Mak11) with a FP-fluorophore.
7. Ensure that the tagged protein continues to perform its normal functions when tagged. The resulting "donor" strains are typically crossed with an "acceptor" strain that does not express the same fluorophore. If comparative kinetics of relocation are to be investigated in single crosses, two distinct proteins can be tagged with different fluorophores, e.g., GFP *vs* mRFP, mCherry or CFP. Both can be expressed in the same cell or they can be expressed in separate cells of different mating type. Transfer thus can be followed in either parallel or anti-parallel fashion. We routinely use centromeric plasmids to express proteins of interest, tag them by covalent integration (Longtine et al., 1998, Janke et al., 2004, Sidebottom et al., 2017, Gardner and Jaspersen, 2014), acquire strains from other labs, or purchase strains (e.g., the GFP collection from Invitrogen/Life Technologies).
8. If the rate of ongoing synthesis and nuclear import make it impossible to focus on copies of the protein that existed before the cross was initiated, use strains in which expression of the tagged protein can be repressed.
9. For this purpose, if (over)expression is from a galactose-inducible *GAL1/10* promoter, pregrow cells in medium with 2% galactose and then transfer them to medium with 2% glucose during the cross. If expression is from a methionine-repressible *MET3* promoter, pregrow cells in medium lacking methionine and then transfer them to complete synthetic medium in which the concentration of methionine is 20 μg/mL (Amberg et al., 2005).

***Note:*** Strains such as those in the commercially available GFP-tagged collection and deletion collection often carry the *met15Δ* deletion (e.g. if they are derived from BY4741/4742). They nevertheless grow in synthetic medium lacking methionine. This may be due to traces of methionine that are said to be present in commercial sources of leucine.

**Figure 1:**
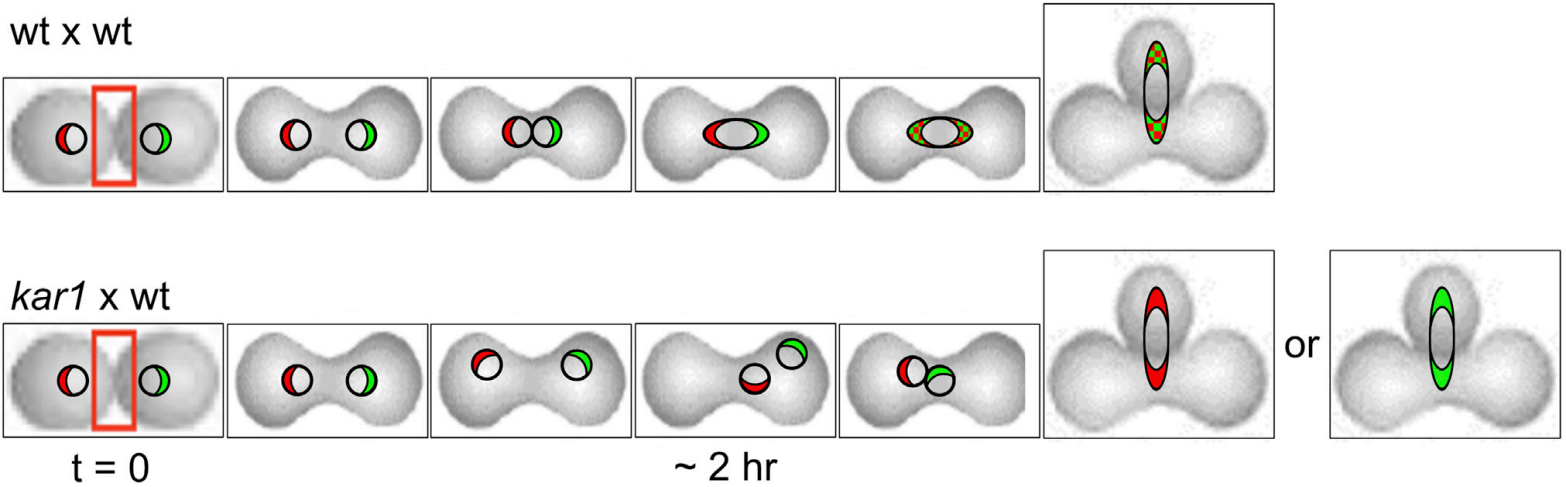
Diagram of steps of cell and nuclear fusion in a [wt x wt] cross and a [*kar1Δ15* x wt] cross In the illustrated time-courses, the first bud forms at a medial site, as is unusually the case (Zapanta Rinonos et al., 2014). The red rectangles indicate the zone of contact at which cell-cell fusion will occur. The distinct nucleolar proteins are represented by different colors. [wt x wt]: Note that the two nucleoli remain well-separated from each other after nuclear fusion, but that the color markers. interchange. [*kar1* x wt]: Note that nuclear fusion is delayed even though the nuclei may contact each other. The two colored nucleolar markers do not intermix. In the final images for the [*kar1* x wt] cross, a single dividing nucleus is illustrated. The second nucleus (although not illustrated) remains in the body of the zygote and divides at the same time (Zapanta Rinonos et al., 2014).

10. To evaluate the possible contribution of new synthesis or export to the cytoplasm followed by reimport, perform control crosses under conditions in which parental nuclei do not fuse. This can be achieved using donor or acceptor strains that carry the *kar1Δ15* mutation in the spindle pole body protein, Kar1. These cells progress normally through the mitotic cell cycle and form zygotes with good efficiency (Vallen et al., 1992). Nevertheless, nuclear fusion is strongly delayed. Moreover, when karyogamy does occur, fusion of spindle pole bodies is blocked, and progeny can be aneuploid (Tartakoff et al., 2018, Dutcher, 1981). Figure 1 schematizes the progress of both [wt x wt] crosses and [*kar1* x wt] crosses.
  a. To introduce the *kar1Δ15* mutation into a *ura3* host transform *ura3* cells ("pop-in") with the integrating *URA3* plasmid pMR1593/pAT1457 (Vallen et al., 1992), after its linearization by cutting within *KAR1* using BglI, followed by column purification according to Qiagen. The Ura+ transformants that are selected have two tandem copies of *KAR1* (wt and mutant) flanking the selective marker. Use lithium acetate for transformation, but other procedures should work well. Use approximately 1/10 of a miniprep to transform a 0.5 mL culture of cells (O.D. = 2).
  b. Grow the transformants in liquid medium under non-selective conditions for 10–20 h.
  c. Plate 0.5 mL of the cell mixture on 5′-FOA plates to select for uracil auxotrophy (*ura-*) that usually results from recombination ("pop-out" events) that excises one copy of *KAR1* (Boeke et al., 1987, Amberg et al., 2005).
    Collect individual colonies with a toothpick.
  d. Grow them as 0.1 mL liquid cultures in preparation of using a bioassay to learn which form of *KAR1* is retained.
    i. Bioassay: Mix 20 μL of the cultures with 10–20 μL from a culture of wildtype cells of the opposite mating type that express a nuclear marker, e.g., Htb2-mRFP (ATY2289). Pipette the entire volume onto the surface of a Petri dish with complete medium.
    ii. After 3–5 h, wash the cells off the plates using 0.5 mL of growth medium and examine them by epifluorescence to evaluate nuclear fusion. Strains of interest characteristically produce zygotes that have two fluorescent nuclei, indicative of the block of karyogamy and implying that they carry the *kar1Δ15* mutation, rather than having a single large nucleus.
      ***Note:*** In this example, the nuclear marker, Htb2-mRFP, routinely becomes present in all nuclei, even if introduced by a single parent.
    iii. Cells that carry the *kar1Δ15* mutation can be frozen at −80°C after mixing with an equal volume of 30% glycerol in growth medium.

### Brightness verification

**Timing: 1–2 days.**

11. With a toothpick, recover a sample of cells stored at −80°C.
12. Transfer into synthetic growth medium and shake the culture.
13. After 10 h, examine the cells in an inverted fluorescent microscope to ensure that the fluorescent nucleolar signal(s) are readily visible. Brightness is critical since cell-cell fusion generally reduces fluorescent intensity by half, as is illustrated below.

### Preparation of petri dishes

**Timing: 3 h.**

14. Autoclave synthetic medium containing 2% agar (Amberg et al., 2005).
15. Pour the medium into Petri dishes.

**CRITICAL:** Leave the plates uncovered under a laminar flow hood for at least one hr at room temperature to allow residual liquid to evaporate, thereby ensuring that the haploid cells plated on them will dry down quickly and therefore be crowded together. If excess moisture remains, mating mixtures plated on them will remain in suspension and zygotes will form with poor efficiency.

**Pause point:** At this point the plates can be stored for up to one week at 10°C.

### Preparation of agarose pads and their use

**Timing: 30 min.**

16. Add Agarose to growth medium (1.5% final).
17. Bring to a boil and vortex.
18. Apply 0.3 mL samples to the surface of a microscope slide that is immediately overlaid with a second slide.

**Pause point:** These slides can be kept in a horizontal position for 1–2 days in a humid slide box in a refrigerator.

19. When the cross has reached an appropriate point for examination, remove the upper slide by gentle horizontal sliding, thereby exposing two surfaces. 0.5–1.0 μL samples of cell pellets can be applied to either or both surfaces.
20. Add a 1 mm (No. 1) coverslip on top of the cells. For time-lapse imaging, seal the coverslip with vaseline to reduce evaporation. The procedure for preparing agarose-pads for imaging is illustrated in (Zapanta Rinonos et al., 2012).

### Cell pre-growth

**Timing: 1 day.**

Mix equal volumes of actively growing cultures. Their final OD_600_ should not exceed 0.5. If possible, adenine should be included in the medium to minimize autofluorescence (see below). As necessary, they can be grown in inducing medium, i.e., using galactose in place of glucose or in methionine-free medium.

## Key resources table

**Table:** 

REAGENT or RESOURCE	SOURCE	IDENTIFIER
**Biological samples**		

*S. cerevisiae* strains and their derivatives	Invitrogen/Thermo Fisher	Yeast Deletion and Yeast GFP Collections
*S. cerevisiae* MAT α Htb2-mRFP	Laboratory stock	ATY2289
*S. cerevisiae* MAT α mRFP-HDEL	Laboratory stock	ATY3196
MAT α *kar1Δ15* mRFP-HDEL	Laboratory stock	ATY6618
MAT a Mak11-GFP Sik1-mRFP	Laboratory stock	ATY8300

**Chemicals, peptides or recombinant proteins**		

Media for cell growth	Sigma	Cold Spring Harbor Yeast Genetics Course Manual (Amberg et al., 2005)
Agarose	Invitrogen/Thermo Fisher	15510-027
Formaldehyde in water, 37%	Fisher Chemicals	F79

**Recombinant DNA**		

pKar2frag-mRFP-HDEL Cut with Xho1 to integrate at *URA3*	(Gao et al., 2005)	pTiKmRFP/pAT1180
ss-GFP-HDEL (*URA3*/CEN)	(Prinz et al., 2000)	pWP1055/pAT1182
Yip5 carrying the *kar1Δ15* mutation Cut with BglII to integrate at *URA3,* then counterselect on 5′-FOA	M. Rose	pMR1593/pAT1457

**Software and algorithms**		

SoftWorX deconvolution software	Applied Precision, Inc.	http://www.sussex.ac.uk/gdsc/intranet/pdfs/softWoRx user manual.pdf

**Other**		

Titer plate shaker	Thermo Fisher	Labline 4625
Sterile toothpicks	Local market	Local market

## Materials and equipment

A Deltavision RT epifluorescence microscope with an automated stage (Applied Precision, Inc) is used for fluorescent imaging with a 100**×** objective.

## Step-by-step method details

### Basic shuttling assays

**Timing: 1 day.**

To follow a tagged protein that is expressed constitutively from its endogenous promoter.

1. Grow two strains of cells (see above), one of which expresses a fluorescent nucleolar protein. One is MAT a and the other is MAT α.
2. Combine aliquots of both parental cells (e.g., 100 μL of each). Vortex.
3. Sediment the cell mixtures and resuspend in synthetic medium with 2% glucose at OD600 ≈ 1.
4. Apply 25 μL samples of these mating mixtures to the surface of Petri dishes containing solid 2% agar in synthetic medium.
5. Leave open in a laminar-flow hood. Liquid should be absorbed in < 15 min. The dishes are then covered. The overall timing of the cross is measured from the moment when the mating mixture is applied to the surface of the Petri dish.

**CRITICAL:** More time can be required for absorption if the volume of the samples is increased. In this event, the experiment will not be suitable for collecting kinetic information. See troubleshooting.

6. After 2 h at room temperature, recover the cell mixture by washing with synthetic medium and gentle pipetting up and down 4–5**×**. Under optimal conditions, many pre-zygotes and zygotes should be visible at this time; however, a majority of haploid cells will remain.

**CRITICAL:** Samples can be vortexed briefly but should not be sonicated. See troubleshooting.

7. Sediment the cells. Without removing the supernatant, recover 0.5–1.0 μL samples of the cell pellet and apply this volume to the surface of freshly-opened Agarose pads, cover with a coverslip, and observe. For imaging in excess of 15 min duration, trim the pads to the dimensions of the coverslip with a single-edge razor blade (after adding the cells) and seal them with vaseline to minimize drying.

**CRITICAL:** Avoid nail polish or mounting media that include organic solvents since they will kill the cells.

8. To restrict attention to relatively early events, we examine zygotes that show no sign of bud emergence that coincides with onset of DNA synthesis (Sena et al., 1976). We then focus on early zygotes that show evidence of nuclear fusion (redistribution of ER/NE markers). In our experience, once transfer of label begins, equilibration is reached within 10–15 min (Tartakoff et al., 2021). As is described below, control experiments need to be performed in parallel.

In these mating protocols, cell encounters, fusion and karyogamy are asynchronous, due to the variable proximity of potential mating partners and the fact that cell fusion can occur only when cells have reached the beginning of the cell cycle (Tartakoff, 2015, Gammie and Rose, 2002). Cells can be presynchronized, e.g., by exposure to α-factor which is then washed away when the two cell types are mixed. Alternatively, one can recover spent medium from cultures of MAT a and MAT α cells and use them to culture cells of the opposite mating type for 1–2 h. In our experience these procedures do not obviously increase the efficiency of zygote formation.

To follow tagged proteins whose expression can be repressed.

9. Pregrow one or both parental cells that expresses a tagged protein of interest from a repressible promoter, using a medium that allows expression (e.g., with galactose or lacking methionine).
10. When the mating mixture is prepared, adjust the composition of the plate used for the cross to repress transcription, e.g., by addition of glucose or methionine. For the many nucleolar proteins that we have studied - judging from the restriction of label to a single nucleolus in [*kar1Δ15* x wt] crosses - the rate of synthesis appears to be sufficiently low so that new synthesis is not a concern over the relevant time period (Tartakoff et al., 2021) - see below.

Regardless of whether protein expression is repressed, intranuclear shuttling can be judged by determining whether the signal(s) that initially was in only one of the cells has become equally visible at both extremities of the elongated zygotic nucleus (i.e., in both of the nucleoli). In [wt x wt] crosses, *cis*-to-*trans* redistribution of nucleolar proteins begins once the two nuclei have contacted each other and in fact can be detected even before their zone of contact has visibly dilated (Tartakoff and Jaiswal, 2009).

According to the goal of the experiments, evaluate possible shuttling either at a single time point or by time-lapse imaging. When only a single time point is needed, the samples can be fixed, as follows:

11. Pipette the mating mixture off the plate with complete medium.
12. Add 1/10 volume of formaldehyde from a 37% stock in water, shaking for 5 min at room temperature.
13. Wash once with growth medium to quench free aldehydes.

**Pause point:** Store samples in water at this point for 1–2 days at 4°C before examination.

### Control experiments

**Timing: 1 day.**

To learn whether any appearance of fluorescent signal in the *trans* nucleus results from continued synthesis or nucleo-cytoplasmic shuttling, we can include two controls:

First control.

14. To learn whether transfer of label occurs before nuclear fusion, include a marker of the lumen of the nuclear envelope (and ER). This is either mRFP-HDEL or GFP-HDEL. The C-terminal HDEL peptide signal in conjunction with its receptor serves to retrieve cargo (mRFP, GFP) to the ER/NE if it reaches the Golgi (Semenza et al., 1990). We find that the "HDEL signal" itself redistributes almost instantaneously upon cell-cell fusion We therefore transform cells with a centromeric plasmid encoding GFP-HDEL (pWP1055/pAT1182)(Prinz et al., 2000), or an integrating plasmid encoding mRFP-HDEL (pTiKmRFP-HDEL/pAT1180) (Gao et al., 2005). We routinely include a HDEL marker in both [wt x wt] and [*kar1* x wt] crosses.

**Figure 2:**
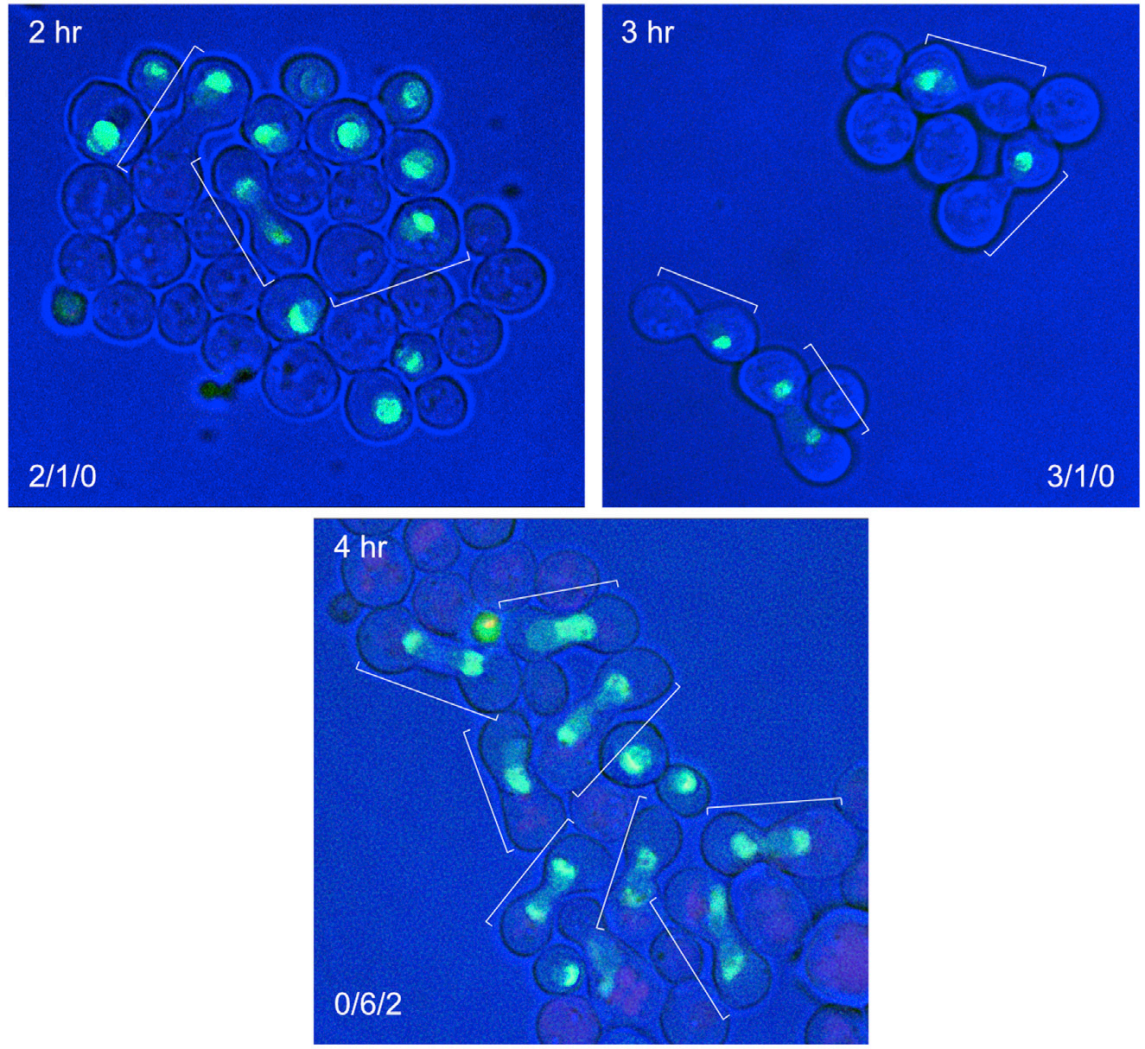
Equilibration of the nucleolar protein, Mak11-GFP, in a [wt x wt] cross Mating mixtures were applied to the surface of synthetic medium Petri dishes for 2, 3 or 4 h at room temperature and then examined. The brackets designate prezygotes and zygotes. The number of zygotes showing no transfer (a), transfer between separate nucleoli (b) or transfer and nucleolar fusion (c) is tabulated in the lower corners of the images. When relocation occurs, both nucleolar crescents become equally labeled. At 4 h, bud emergence is seldom visible. Strains: ATY8300, ATY3196.

Since many ribosome assembly factors, in addition to being concentrated in the nucleolus, are present at low levels throughout the nucleoplasm or actually line the nuclear envelope (Tartakoff et al., 2021), *cis-trans* continuity can often be evident without including an additional label (Figure 2).

Second control.

**Figure 3:**
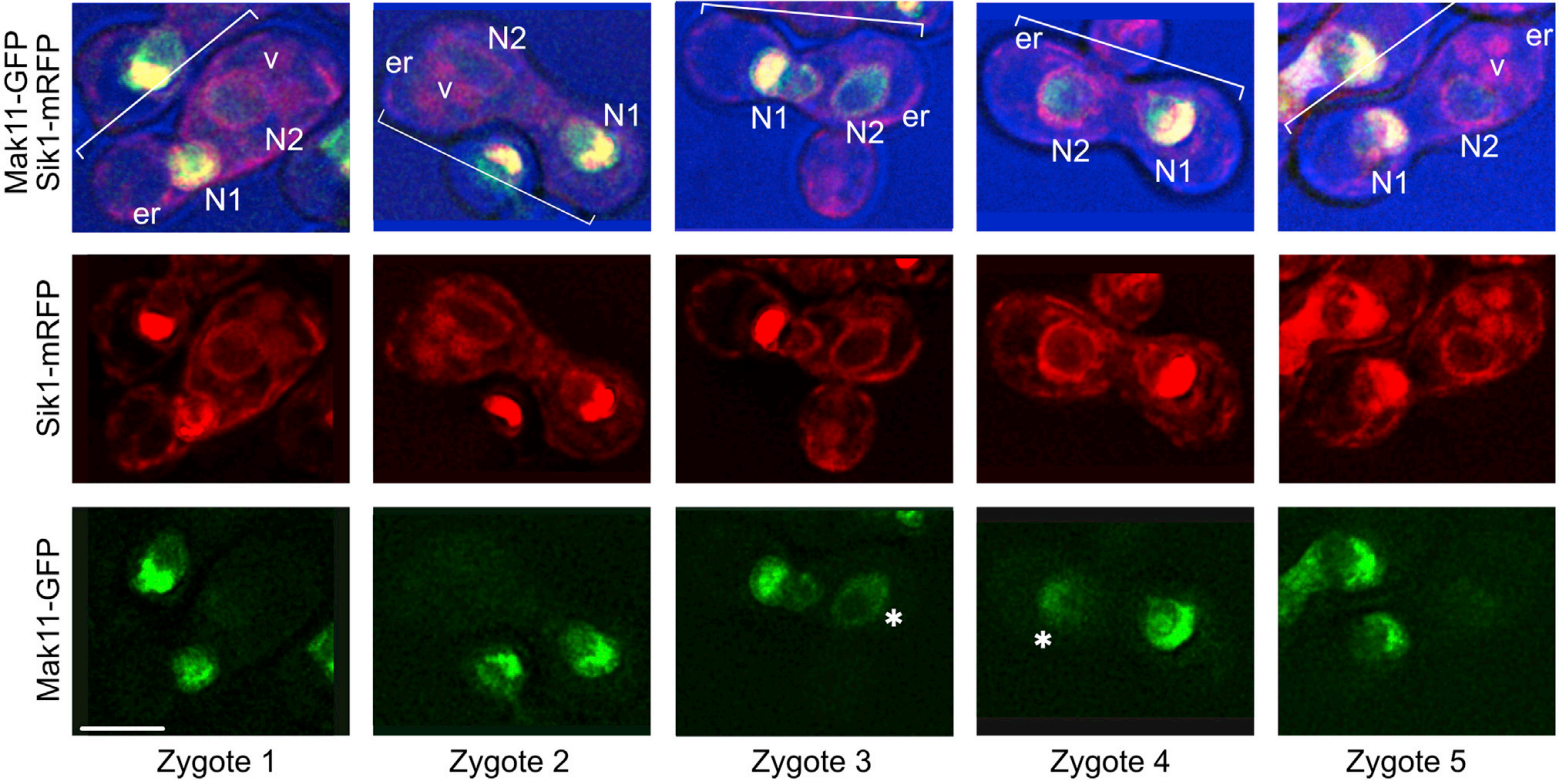
Representative observations from a [*kar1Δ15* x wt] cross after 4 h One of the parental cells expressed Mak11-GFP and Sik1-mRFP, while the other expressed mRFP-HDEL. The pairs of nuclei are encircled by the mRFP-HDEL signal that also highlights the ER at the periphery of each cell. In each zygote, one nucleus shows the strong Mak11-GFP and Sik1-mRFP signals. The other nucleus is either unlabeled or is labeled much more weakly as designated (∗). The scale bar is 5 microns. er: endoplasmic reticulum, N1/N2: nuclei, v: vacuole.

15. Judge the possible contribution of nucleocytoplasmic shuttling and new synthesis by following GFP-tagged AFs in crosses with a *kar1Δ15* mutant, as described above. None of the dozens of nucleolar proteins that we have studied show labeling of both nuclei in such dikaryons over 2.5 h (Tartakoff et al., 2021) - Figure 3.

### Imaging

**Timing: 10 min–2 h**

The number of early zygotes that can be imaged in a single field is difficult to control.

16. Collect images of 3−10 fields and examine at least 25 zygotes per cross.
17. Use a Deltavision RT epifluorescence microscope with an automated stage (Applied Precision, Inc) and an oil immersion objective (Olympus UPlanApo 100**×**/1.40; ∞/0.17/FN26.5).
18. Capture z-stacks at 0.5–1.0 μm intervals using a CCD digital camera (Photometrics CoolSnap HQ). % transmission = 100%, exposure time 0.05–1 s, field 1040**×**1040 pixels, binning 1**×**1.
19. Examine through-focal series spanning a total of 6 μ in all cases.
20. Remove out-of-focus light using the Softworks deconvolution program (http://www.sussex.ac.uk/gdsc/intranet/pdfs/softWoRx user manual.pdf).
21. Repeat experiments 2–4 **×**. See troubleshooting.

## Expected outcomes

To evaluate intranuclear shuttling we initiate crosses on synthetic medium plates and either let the cross proceed for 2 h and then examine the cell mixture immediately, or - alternatively - begin time-lapse examination. In this case, we typically examine at least 25 such zygotes and score them with regard to *cis-trans* equilibration of signal. The distribution of the zygotes in the image field will depend on their concentration in the sample that was applied to the Agarose pad. If too concentrated, they overlap and it is difficult to have multiple zygotes (or even both ends of the same zygote) in a single focal plane.

Time-lapse allows one to witness early stages of prezygote formation, as well as progression. In such experiments we find that the t_1/2_ for Mak11-GFP and Sik1-mRFP equilibration is 5–15 min after nuclear fusion (Tartakoff et al., 2021). The presumably anoxic conditions under the coverslip are not obviously problematic, judging from the continued nuclear fusion and budding.

According to the time of incubation prior to being applied to the slide, a variable fraction of the zygotes will have progressed through the classic stages of 1) shmoos with a polarized nucleus (in which the spindle pole body faces the shmoo tip and the nucleolus is at the opposite extremity), 2) prezygotes (in which a flat interface has been established between the apices of both cells), 3) early "linear" zygotes (in which the nuclei make symmetric contact at their apices), 4) later "linear" zygotes (in which nuclei have fused together but there still is no sign of bud emergence), and 5) mature zygotes (in which the nucleoli have merged together and there are single buds at various stages of growth). Zygotes bud repeatedly, as do haploid and diploid cells (Stone et al., 2000, Zapanta Rinonos et al., 2014, Tartakoff, 2015, Ydenberg and Rose, 2008).

## Troubleshooting

### Problem 1

Cells do not form many zygotes.

### Potential solution

The most important consideration is that the parental cells are growing rapidly (mid-log phase or more dilute) when they are crossed. Even if they have not reached saturation density, it is important to dilute them 5–10**×** into fresh medium and shake the cultures for 3–4 h before initiating a cross. It is not advisable to initiate cultures from stored samples and study them without 10 h growth, although the presence of dead bystander cells does not obviously interfere with zygote formation.

A major consideration is the speed with which the mating mixtures are absorbed into the surface of Petri dishes containing growth medium. If more than 30 min is required, plates should be dried for a longer period of time than the standard 1 h.

### Problem 2

Endogenous fluorescence is strong.

### Potential solution

Cells with lesions along the adenine biosynthetic pathway (*ade2* or *ade3* mutants) accumulate fluorescent pigments in their vacuoles and this can interfere with imaging. The problem is most pronounced if cells approach saturation. In general, the vacuolar signal becomes minimal if adenine is added to 200 μg/mL during cell growth.

### Problem 3

Photobleaching.

### Potential solution

Especially the "mRFP-HDEL" signal becomes photobleached during time-lapse imaging. If it is used, it therefore is generally inadvisable to collect z-stacks of images at less than 15 min intervals.

### Problem 4

Foreign cells.

### Potential solution

Crosses should be made between two clonal populations of cells. If any other cells are seen, the parental cultures should be recloned by "streaking to singles." This is achieved by using three successive toothpicks to streak the cells orthogonally onto complete medium in Petri dishes. The streaking results in an array of colonies since the first streak is horizontal at the top of the plate, the second streaks are vertical (intersecting the initial streak), and the third back-and-forth streaks are again horizontal, intersecting the second streaks. The plate is then incubated 1–2 days. When colonies appear, grow up several clones, test them individually, and verify their genotypes.

### Problem 5

Zygotes are not in a single image plane.

### Potential solution

In recovering the mating mixtures from Petri dishes, a certain amount of agar is also recovered. This can make it difficult for the coverslip to be horizonal, In this event, we recommend using Agarose in place of agar for the cross itself. It is also possible to conduct crosses "under medium" by allowing mating mixtures in 0.1–0.5 mL to settle and form a sub-confluent monolayer at the bottom of a plastic well in a multi-well plate, which is then left immobile (Zapanta Rinonos et al., 2014).

Optical uniformity can also be improved by being careful not to apply an excessive number of cells to the Agarose pads used for visualizaton..

## Quantification and statistical analysis

The lack of synchrony makes certain types of quantitative analysis problematic. Quantitation can be achieved by counting the number of early zygotes that show equilibration of the labeled protein; however, we find that the results are already very clear-cut at a qualitative level when the labeled proteins do (or do not) redistribute to the two ends of the zygote. This is meaningful since in the [*kar1* x wt] control crosses - even when the incubations are prolonged - there is little or no redistribution (Figure 3). An alternative quantitative protocol could use time-lapse imaging; however, this requires that the zygotes tolerate imaging for 1–3 h. A complicating consideration is that progressive photobleaching will occur.

## Limitations

Assays that depend on zygote formation are inappropriate for certain yeast strains. For example, the remodeling of the cell surface that is intrinsic to establishment of *cis-trans* continuity during zygote formation requires control of cell wall integrity to ensure protection against osmotic stress. A variety of yeast mutants cannot exert this control and therefore are not suitable for efficient formation of normal zygotes (Tartakoff, 2015, Aguilar et al., 2007, Philips and Herskowitz, 1997). Indeed, even crosses of wildtype cells show occasional evidence of incomplete fusion or what appears to be osmotic rupture.

## Resource availability

### Lead contact

Further information and requests for resources and reagents should be directed to and will be fulfilled by the lead contact, Dr. A.M.Tartakoff, amt10@case.edu.

### Materials availability

This study did not generate new unique reagents.

### Data and code availability

This study did not generate datasets*.*
